# Evaluation of MMR Status and PD-L1 Expression Using Specimens Obtained by EUS-FNB in Patients with Pancreatic Ductal Adenocarcinoma (PDAC)

**DOI:** 10.3390/diagnostics12020294

**Published:** 2022-01-25

**Authors:** Alina Constantin, Vlad Iovănescu, Irina Mihaela Cazacu, Bogdan Silviu Ungureanu, Cătălin Copăescu, Cezar Stroescu, Nona Bejinariu, Adrian Săftoiu

**Affiliations:** 1 Department of Gastroenterology, Ponderas Academic Hospital, 014142 Bucharest, Romania; adriansaftoiu@aim.com; 2 Research Center of Gastroenterology and Hepatology Craiova, University of Medicine and Pharmacy of Craiova, 200349 Craiova, Romania; iovanescu_vlad@yahoo.com (V.I.); irina.cazacu89@gmail.com (I.M.C.); bogdan.ungureanu@umfcv.ro (B.S.U.); 3 Faculty of Medicine, Titu Maiorescu University, 040441 Bucharest, Romania; 4 Department of Surgery, St. Mary Hospital, 011172 Bucharest, Romania; cezar.stroescu@gmail.com; 5 Santomar Oncodiagnostic, Regina Maria Histopathology Laboratory, 400350 Cluj Napoca, Romania; nonarebei@yahoo.com

**Keywords:** pancreatic ductal adenocarcinoma, endoscopic ultrasound-guided fine-needle biopsy, PD-L1, MMR expression

## Abstract

Deficient DNA mismatch repair status (dMMR)/high microsatellite instability have been shown to be predictive biomarkers for immune checkpoint inhibitor drugs which block the programmed death protein-1/programmed death ligand-1 (PD-1/PD-L1) interaction between tumor cells and activated T cells. The aim of this study was to determine the prevalence of MMR status and quantification of PD-L1 expression in pancreatic endoscopic ultrasound-guided fine-needle biopsy (EUS FNB) specimens. Immunochemistry (IHC) was performed on consecutive archived treatment-naïve formalin-fixed paraffin-embedded EUS-FNB samples. The specimens were considered to have PD-L1 expression if PD-L1 was expressed in ≥1% of tumor cells and a high level of expression if ≥50%. Tumors with absent nuclear staining of DNA mismatch repair proteins (MLH1, MSH2, MSH6, or PMS2) were classified as dMMR. A total of 28 treatment-naïve patients who underwent EUS-FNB and had a final diagnosis of pancreatic ductal adenocarcinoma (PDAC) were included in the study. All the EUS-FNB samples were adequate for the evaluation of MMR and PD-L1 expression. None of the patients with PDAC included in the study had a dMMR tumor. PD-L1 expression was identified in 39% of the cohort (*n* = 11). Expression thresholds of ≥1%, ≥10%, and ≥50% in tumor cells were identified in 11 (39%), 4 (14%), and 1 (4%) patients, respectively. The evaluation of MMR status and PD-L1 can be successfully performed on EUS-FNB pancreatic specimens. Furthermore, MMR expression failed to show utility in recognizing immunotherapy vulnerability in pancreatic cancer; the only recommendation for testing remains for patients with heritable cancers. Meanwhile high PD-L1 expression was correlated with poor prognosis. This association may identify a subgroup of patients where immune checkpoints inhibitors could provide therapeutic benefits, spotlighting the role of EUS-FNB in the field of immune-oncology.

## 1. Introduction

Pancreatic ductal adenocarcinoma (PDAC) is one of the most aggressive malignancies and has a dismal prognostic. Despite radical surgery and chemotherapy regimens, the median survival for PDAC is quantified in months, and the 5-year survival rate is approximately 9%, only slightly changed in the past three decades [1].

Programmed cell death protein I (PD-1) and its ligand, programmed death ligand 1 (PD-L1), represent regulatory corresponding receptors on the membrane of infiltrating immune cells and tumor cells, respectively. The binding of PD-1 to PD-L1 leads to suppression of T cell activation and consequently helps tumor cells to evade immune surveillance [2]. Furthermore, the PD-1/PD-L1 pathway has been extensively studied regarding the correlation with cancer progression and clinical outcomes. Recently, inhibition of PD-1/PD-L1 with therapeutic antibodies showed broad efficacy in several cancers such as advanced melanoma, non-small lung cancer, colorectal cancer, and renal cell carcinoma [3,4]. Particularly, tumors with deficient DNA mismatch repair (MMR) and microsatellite instability (MSI-H) have a predictive increased response to immune checkpoint inhibitors. Beyond that, Vanderwalde and colleagues highlighted in a recent study a strong correlation between the frequency of dMMR/MSI-H and PD-L1 expression in various malignancies including endometrial cancer, gastric adenocarcinoma, and colorectal cancer. However, the frequency of MSI-H was shown to be 3% in a study including 11,348 patients with solid tumors. Meanwhile, dMMR in 833 surgically resected pancreatic cancer specimens was found only in 0.3% cases and it was associated exclusively with Lynch Syndrome [5].

In 2017, Pembrolizumab, an anti-PD-L1 molecule was approved by the US Food and Drug Administration (FDA) for patients with unresectable or metastatic d-MMR solid tumors after five clinical trials with promising results—partial or complete response obtained in 39.6% of patients inroled in that studies [6]. It was the first time that the FDA approved a treatment for cancer based merely on tumors’ biomarker status.

The aim of the current study was to determine whether endoscopic ultrasound fine needle biopsy (EUS-FNB) provides sufficient material in order to determine both PD-L1 and dMMR status. Moreover, we sought to evaluate the prognostic value of MSI status and PD-L1 expression and to compare our results obtained through EUS-FNB with data from literature regarding resected pancreatic tumors specimens.

## 2. Patients and Methods

### 2.1. Patients

**Figure 1:**
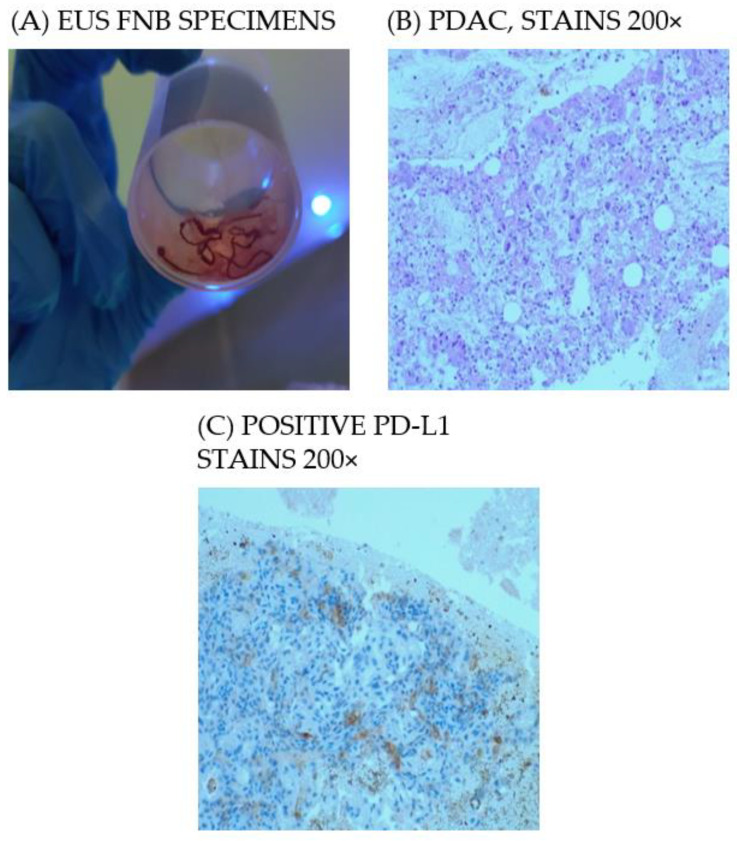
(**A**) EUS FNB specimens; (**B**) PDAC, HE stains 200×; and (**C**) Positive PD-L1 stains 200×.

Between 2019–2020, 28 consecutive patients with a suspicion of pancreatic masses underwent EUS-FNB, Contrast Enhanced Harmonic EUS (CH-EUS), and EUS elastography for confirmation of diagnosis. Inclusion criteria were women or men aged 18 to 90 years old; signed informed consent for EUS and EUS-FNB; the diagnosis of adenocarcinoma confirmed histologically by fine needle biopsy; and a resectable, unresectable, locally advanced, and/or metastatic disease. However, patients with previous radiotherapy or chemotherapy were excluded from analysis. A positive histopathological diagnosis has been taken as a proof of malignancy (Figure 1B). Thus, IHC staining for PD-L1 and dMMR was performed on EUS-FNB malignant specimens. Furthermore, patients were followed for a minimum period of 12 months through clinical examination. This study obtained approval from the hospital’s institutional review board for human research and is registered on clinicaltrials.gov (NCT03820921).

### 2.2. EUS–FNB Technique for the Pathological Diagnosis

We performed EUS using a convex linear-array endoscope (EG-3870UTK, Pentax Europe Gmbh, Hamburg, Germany) connected to an ultrasonography machine (Arieta V70, Hitachi, Zug, Switzerland) in order to make a station-based approach examination of the pancreas. Tumor characteristics were described (size, echogenicity, and echostructure,) as well as presence/absence of power Doppler signal. EUS-FNB was made using a fanning technique, performing at least 2 passes in the absence of an onsite cytopathologist, with a 22-gauge needle (Acquire FNB or Expect FNA Boston Scientific, Marlborough, MA, USA). By reinserting a stylet into the FNB needle, aspirated samples were placed on a glass slide for cytological examination (Papanicolau method) and on a recipient with 10% paraformaldehyde for histological evaluation (Figure 1A).

### 2.3. IHC

**Figure 2:**
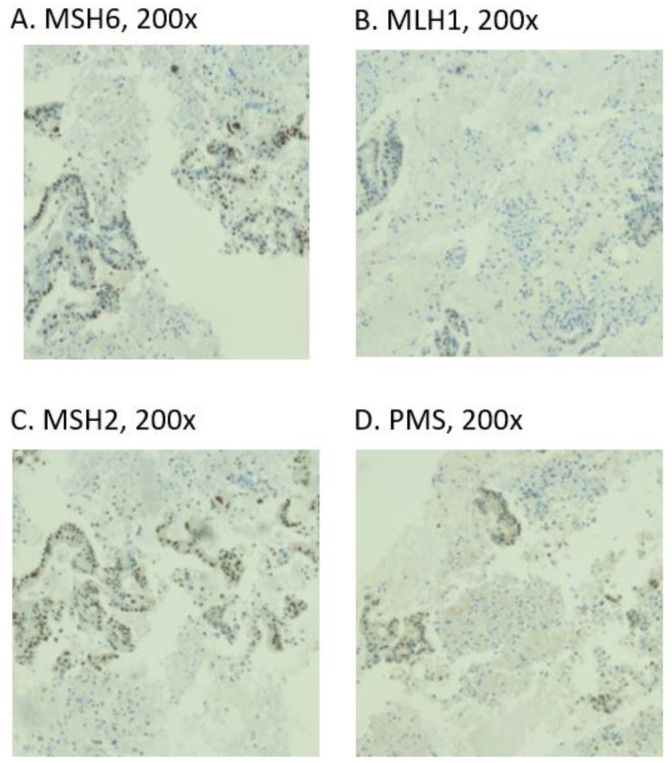
IHC staining for the MMR proteins.

EUS-FNB specimens obtained from treatment-naïve patients were archived respecting formalin fixation and paraffin embedding protocols and were subjected to IHC staining. Briefly, Ventana BenchMark Ultra automated slide-staining system was used in order to stain 4 μm thick tissue sections utilizing the following antibodies: anti-PD-L1 (clone SP263 Ventana), MLH1 (clone ES05 Leica), MSH2 (clone G219-1129, Ventana), MSH6 (clone SP93 Novus Biological), and PMS2 (clone A16-4, Ventana). Diaminobenzidine was used as a chromogen for UltraView detection in order to visualize antigen–antibodies reactions. A specimen was considered adequate for evaluation only if a minimum of 100 tumor viable cells were found on the slides. Membranous staining was the hallmark for positive PD-L1 expression (Figure 1C). The tumor proportion score (TPS) has been calculated as the percentage of viable tumor cells with complete or partial membrane staining at any intensity. PD-L1 expression was considered in specimens with TPS > 1% and high PD-L1 expression was considered in patients with TPS > 50%. In the case of absent nuclear staining of DNA mismatch repair protein (PMS2, MSH2, MSH6, or MLH1), the tumor was targeted as dMMR (Figure 2).

## 3. Results

### 3.1. Patient Characteristics

Twenty-eight treatment naïve patients with pancreatic ductal adenocarcinoma diagnosed through EUS-FNB were subjected to IHC tests in order to assess adequacy for MMR status and PD-L1 expression testing. The characteristics of the 28 patients enrolled in the study are summarized in Table 1.

The median age of the overall cohort was 63.9 (range 43–83), 52% were male, and the survival rate at 12 months following EUS diagnosis was 21.4%. Furthermore, the median tumor size was 35.8 (range 16–60 mm). Interestingly, 80% of patients had stage ≥ III according to 8th AJCC classification [7] at the moment of diagnosis.

### 3.2. EUS FNB for the Pathological Diagnosis

The median number of needle passes was three (IQR 2–5), and needle used for FNB was 22G Acquire Boston Scientific in 25 (89%) patients and 22G Expect Boston Scientific in 3 (11%) patients.

### 3.3. MMR Status and PD-L1 Expression

**Figure 3:**
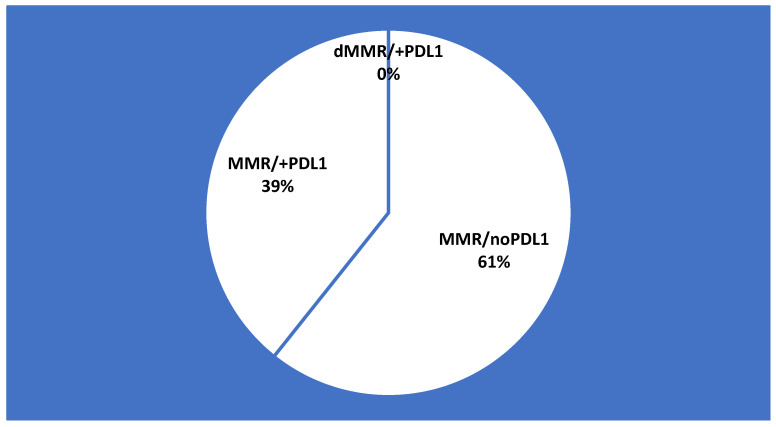
MMR and PD-L1 prevalence in the study cohort.

**Figure 4:**
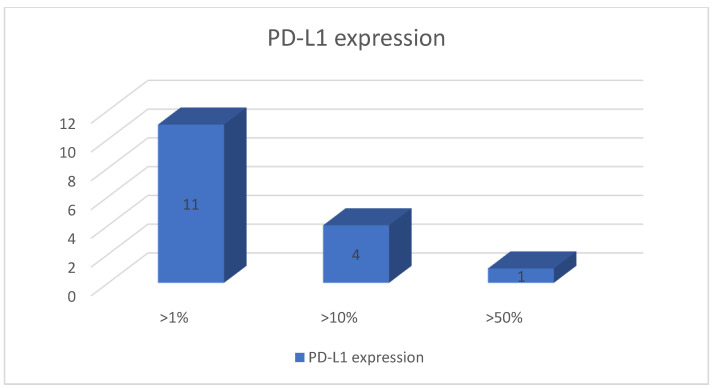
Quantification of PD-L1 expression positivity for EUS-FNB specimens of treatment-naïve PDAC.

All the specimens were adequate (100%) in order to assess MMR and PD-L1 status by IHC. Thus, DNA mismatch repair proteins (PMS2, MSH2, MSH6, and MLH1) exhibited present nuclear staining in all specimens. In other words, deficient MMR was not identified among our cohort (Figure 3). On the other hand, the thresholds for PD-L1 expression >1%, >10%, and >50%were identified in 11 (39%), 4 (14%), and 1 (4%) patients, respectively (Figure 4).

### 3.4. Immunotherapy Eligibility According to Biomarkers

Based on FDA recommendation regarding approved tumor-agnostic immunotherapy, pembrolizumab had no indication criteria in our cohort according to dMMR and PD-L1 status. Instead, tumors with PD-L1 expression more than 10% (18% of patients) may be sensitive to combined immune checkpoint inhibitors [8,9].

## 4. Discussion

Among our PDAC patient cohort we have determined that EUS FNB can successfully evaluate MMR status and PD-L1 expression by IHC. Furthermore, samples obtained by EUS-FNA/FNB have widespread potential for molecular analysis with the aim of assessing a precise diagnosis and prognosis of PDAC. Molecular analysis of the EUS-FNB samples such as KRAS testing [10] to improve positive or differential diagnosis in PDAC is also dependent on the quality of the material obtained through EUS.

According to the results of a recent meta-analysis, it has been established that FNB needles in solid pancreatic tumors were associated with better diagnostic yield than FNA needles in the absence of ROSE, with a lower number of needle passes needed [11]. Although tissue specimens were useful for genotyping using next generation sequencing (NGS), IHC, or establishing tumor organoids, cytology smears were superior to FNB specimens in terms of overall cellularity, sequencing metrics, or tumor fraction [12,13]. Thus, the objective dictates the needle type to be used. In our study, we used FNB needles with a median of three needle passes obtaining 100% diagnostic accuracy rate and 100% overall success rate of the IHC. As a comparison, in two studies diagnostic accuracy rate on histology using FNA needles was 79.7% and 69.3%, respectively [14,15].

The positive rate of PD-L1 with a cutoff value of 10% in surgical specimens using IHC staining ranged in the literature from 22% to 39% [16,17,18]. Therefore, the rate of PD-L1 positive cases from our study (18%) is comparable with previous results taking into account that our specimens are obtained through FNB, having small fragments available for analysis, not the entire resection piece. The fanning technique may be useful to obtain an adequate accuracy rate considering that PD-L1 is distributed in a patchy manner.

PD-L1 expression in surgical specimens has been found to be a predictor of poor prognosis in PDAC patients in various studies. [16,17,18]. In our cohort, PD-L1 positive patients with stage IV cancer at the moment of diagnosis were in slightly greater numbers than PD-L1 negative patients with the same stage of disease (66.6% vs. 57%). However, the PD-L1 positive and PD-L1 negative patients showed a slightest difference regarding survival analyses (27.2% vs. 17.6% in terms of 1 year disease specific survival) [19,20].

In the era of precise immuno-oncology, endosonographers are required to improve their technique and use the right needle in order to provide a quality material for ancillary molecular analysis. A satisfactory specimen with cytologic adequacy allows the identification of prognosis or chemosensitivity predictors. Thus, the latest studies include combined therapies for metastatic disease in order to improve PDAC dismal prognosis. Electro immunotherapy was proposed to establish a durable and effective anti-tumor response [21]. Moreover, preclinical results showed an efficacity in targeting PD-L1 in combination with IL-6 [8], CCL5 [9], or radiotherapy [22]. Although PDAC has a low mutational burden and a cold non-immunogenic environment, the barriers have been already overcome and progress has been made.

It is noteworthy that PD-L1 and MMR/MSI status was evaluated until now in surgically resected specimens, with the exception of only one small study that followed PD-L1 and MMR status in EUS-FNB specimens from patients with PDAC and pancreatic metastases [23]. We have to take into account that resected specimens come from patients with less advanced disease. Therefore, our study distinguishes by his uniform cohort that exclusively included patients with pancreatic adenocarcinoma not only in the phases where surgical resection was recommended, but also in the advanced stages before any neoadjuvant treatment. These tests are recommended to be performed on patients with advanced disease and before any neoadjuvant treatment having in mind that radio-chemotherapy may influence MMR and PD-L1. Regarding the limitations of the study, the small sample size may influence the percentage of PD-L1 obtained through fine needle biopsy. Targeting different areas of the tumor by using fanning technique may improve the results. Another limitation was the small number of patients enrolled in the study. Further multicenter studies are needed in order to target various combinations of immune checkpoints inhibitors.

## 5. Conclusions

To sum up, the evaluation of MMR status and PD-L1 can be successfully performed on EUS-FNB pancreatic specimens. Furthermore, MMR expression failed to show utility in recognizing immunotherapy sensibility in pancreatic cancer, the only recommendation for testing remaining for patients with hereditary cancers. The rate of PD-L1 with a cutoff value of 10% was similar with previous studies performed on PDAC resected specimens. Although the status of PD-L1 showed no influence regarding survival analyses, high PD-L1 expression was correlated with poor prognosis. Therefore, this association may identify a subgroup of patients where immune checkpoints inhibitors could provide therapeutic benefits, spotlighting the role of EUS-FNB in the field of personalized medicine.

## Figures and Tables

**Table 1:** Patient Characteristics.

Parameter	Number
Age, median (range) (years)	63.9 (43–83)
Sex, male *n*%	13 (52)
Tumor size, median (IQR) (mm)	35.8 (16–60 mm)
Stage (AJCC classification) I/II/III/IV	4/16/20/60
1 year survival rate PD-L1 positive/PD-L1 negative	21.4 (6) 33.3/66.6

## Data Availability

All data supporting the study can be located in the archive of Santomar Oncodiagnostic, Regina Maria Histopathology Laboratory, 400350 Cluj Napoca, Romania.

## References

[1] Siegel R.L., Mph K.D.M., Jemal A. (2018). Cancer statistics. CA Cancer J. Clin..

[2] Sanmamed M., Chen L. (2014). Inducible expression of B7-H1 (PD-L1) and its selective role in tumor site immune modulation. Cancer J..

[3] Brahmer J.R., Tykodi S.S., Chow L.Q., Hwu W.J., Topalian S.L., Hwu P., Drake C.G., Camacho L.H., Kauh J., Odunsi K. (2012). Safety and activity of anti–PD-L1 antibody in patients with advanced cancer. N. Engl. S. Afr..

[4] Wang X., Bao Z., Zhang X., Li F., Lai T., Cao C., Chen Z., Li W., Shen H., Ying S. (2017). Effectiveness and safety of PD-1/PD-L1 inhibitors in the treatment of solid tumors: A systematic review and meta-analysis. Oncotarget.

[5] Hu Z., Shia J., Stadler Z., Varghese A., Capanu M., Salo-Mullen E., Lowery M.A., Diaz L.A., Mandelker D., Yu K.H. (2018). Evaluating mismatch repair deficiency in Pancreatic Adenocarcinoma: Challenges and recommendations. Clin. Cancer Res..

[6] (2017). US Food and Drug Administration approves first cancer treatment for any solid tumors with specific biomarker. Cancer.

[7] Amin M.B., Edge S., Greene F., Byrd D.R., Brookland R.K., Washington M.K., Gershenwald J.E., Compton C.C., Hess K.R. (2017). AJCC Cancer Staging Manual.

[8] Mace T.A., Shakya R., Pitarresi J.R., Swanson B., McQuinn C.W., Loftus S., Nordquist E., Cruz-Monserrate Z., Yu L., Young G. (2018). IL-6 and PD-L1 antibody blockade combination therapy reduces tumour progression in murine models of pancreatic cancer. Gut.

[9] Wang X., Li X., Wei X., Jiang H., Lan C., Yang S., Wang H., Yang Y., Tian C., Xu Z. (2020). PD-L1 is a direct target of cancer-FOXP3 in pancreatic ductal adenocarcinoma (PDAC), and combined immunotherapy with antibodies against PD-L1 and CCL5 is effective in the treatment of PDAC. Signal Transduct. Target. Ther..

[10] Bournet B. (2014). Role of endoscopic ultrasound in the molecular diagnosis of pancreatic cancer. World J. Gastroenterol..

[11] Khan M., Grimm I., Ali B., Nollan R., Tombazzi C., Ismail M., Baron T. (2017). A meta-analysis of endoscopic ultrasound–fine-needle aspiration compared to endoscopic ultrasound–fine-needle biopsy: Diagnostic yield and the value of onsite cytopathological assessment. Endosc. Int. Open.

[12] Lee B., Cho C., Jung M., Jang J., Bae H. (2017). Comparison of histologic core portions acquired from a core biopsy needle and a conventional needle in solid mass lesions: A prospective randomized trial. Gut Live.

[13] Iacobuzio-Donahue C., Ryu B., Hruban R., Kern S. (2002). Exploring the host desmoplastic response to pancreatic carcinoma. Am. J. Pathol..

[14] Yoshizawa N., Yamada R., Sakuno T., Inoue H., Miura H., Takeuchi T., Nakamura M., Hamada Y., Katsurahara M., Tanaka K. (2018). Comparison of endoscopic ultrasound-guided fine-needle aspiration and biopsy with 22-gauge and 25-gauge needles for the “precision medicine” of pancreatic cancer. Medicine.

[15] Matsumoto K., Ohara T., Fujisawa M., Takaki A., Takahara M., Tanaka N., Kato H., Horiguchi S., Yoshida R., Umeda Y. (2019). The relationship between the PD-L1 expression of surgically resected and fine-needle aspiration specimens for patients with pancreatic cancer. J. Gastroenterol..

[16] VanderWalde A., Spetzler D., Xiao N., Gatalica Z., Marshall J. (2018). Microsatellite instability status determined by next-generation sequencing and compared with PD-L1 and tumor mutational burden in 11,348 patients. Cancer Med..

[17] Nomi T., Sho M., Akahori T., Hamada K., Kubo A., Kanehiro H., Nakamura S., Enomoto K., Yagita H., Azuma M. (2007). Clinical Significance and Therapeutic Potential of the Programmed Death-1 Ligand/Programmed Death-1 Pathway in Human Pancreatic Cancer. Clin. Cancer Res..

[18] Lie J.W., Lu Y., Shen G.J. (2016). The relationship of B7-H1 with clinicopathological characteristics and prognosis of pancreatic carcinoma. Chin. J. Gen. Pract..

[19] Tessier-Cloutier B., Kalloger S.E., Al-Kandari M., Milne K., Gao D., Nelson B.H., Renouf D.J., Sheffield B.S., Schaeffer D.F. (2017). Programmed cell death ligand 1 cut-point is associated with reduced disease specific survival in resected pancreatic ductal adenocarcinoma. BMC Cancer.

[20] Gao H.-L., Liu L., Qi Z.-H., Xu H.-X., Wang W.-Q., Wu C.-T., Zhang S.-R., Xu J.-Z., Ni Q.-X., Yu X.-J. (2018). The clinicopathological and prognostic significance of PD-L1 expression in pancreatic cancer: A meta-analysis. Hepatobiliary Pancreat. Dis. Int..

[21] Geboers B., Timmer F., Ruarus A., Pouw J., Schouten E., Bakker J., Puijk R., Nieuwenhuizen S., Dijkstra M., Tol M.V.D. (2021). Irreversible electroporation and nivolumab combined with intratumoral administration of a toll-like receptor ligand, as a means of in vivo vaccination for metastatic pancreatic ductal adenocarcinoma (PANFIRE-III). A phase-I study protocol. Cancers.

[22] Azad A., Lim S.Y., D’Costa Z., Jones K., Diana A., Sansom O.J., Kruger P., Liu S., McKenna W.G., Dushek O. (2017). PD -L1 blockade enhances response of pancreatic ductal adenocarcinoma to radiotherapy. EMBO Mol. Med..

[23] Gleeson F.C., Levy M.J., Roden A.C., Boardman L.A., Sinicrope F.A., McWilliams R.R., Zhang L. (2018). EUS fine-needle pancreatic core biopsy can determine eligibility for tumor-agnostic immunotherapy. Endosc. Int. Open.

